# Emergence of *bla*_NDM–1_-carrying *Enterobacter chengduensis* in China

**DOI:** 10.3389/fmicb.2024.1404996

**Published:** 2024-08-14

**Authors:** Hongyu Fu, Zhichen Zhu, Xiao Wang, Jingnan Lv, Jie Zhu, Liang Chen, Hua Yu, Hong Du

**Affiliations:** ^1^Department of Clinical Laboratory, The Second Affiliated Hospital of Soochow University, Suzhou, Jiangsu, China; ^2^Department of Blood Transfusion, The Children’s Hospital of Soochow University, Suzhou, Jiangsu, China; ^3^MOE Key Laboratory of Geriatric Diseases and Immunology, The Second Affiliated Hospital of Soochow University, Suzhou, Jiangsu, China; ^4^Sichuan Provincial Key Laboratory for Human Disease Gene Study, Department of Laboratory Medicine, Sichuan Provincial People’s Hospital, University of Electronic Science and Technology of China, Chengdu, China; ^5^Department of Pharmacy Practice, School of Pharmacy and Pharmaceutical Sciences, University at Buffalo, Buffalo, NY, United States

**Keywords:** *Enterobacter chengduensis*, carbapenemase, *bla*_NDM–1_, ST414, IncC

## Abstract

**Introduction:**

*Enterobacter chengduensis* was defined as a novel species in the genus. *Enterobacter* in 2019, however, antimicrobial resistance, such as carbapenem resistance, has rarely been described in *E. chengduensis*. This study described the molecular features of four carbapenem-resistant *E. chengduensis* strains collected from a tertiary health care hospital in Southwest China.

**Methods:**

Whole genome sequencing (WGS) was used to determine the genome sequence of four *E. chengduensis* strains. The precise species of strains were identified by average nucleotide identity (ANI) and *in silico* DNA-DNA hybridization (isDDH). The clonal relatedness of four *E. chengduensis* strains and additional 15 ones from NCBI were examined through phylogenetic analysis. The molecular features of *E. chengduensis* and genetic structure of carbapenemase- encoding plasmids were characterized through genomic annotation and analysis.

**Results:**

The results revealed the emergence of *bla*_NDM–1_-carrying *E. chengduensis* strains in China. Multilocus sequence typing (MLST) analysis showed that all 19 *E. chengduensis* belonged to the same sequence type of ST414. Core SNP analysis suggested the potential intrahospital clonal transmission of ST414 *E. chengduensis*. The carbapenemase-encoding gene *bla*_NDM–1_ was harbored by an IncC-type plasmid, which was experimentally confirmed to be able to conjugate.

**Discussion:**

This study reports the first emergence and potential clonal transmission of *bla*_NDM–1_-carrying *E. chengduensis*. Further surveillance should be advocated to monitor the dissemination of carbapenem-resistant *E. chengduensis* and *bla*_NDM–1_-harboring IncC-type plasmids in China.

## Introduction

As the third most prevalent pathogenic species of Enterobacteriaceae in humans, after *Escherichia* and *Klebsiella*, the *Enterobacter* genus can trigger various clinical infections, including bloodstream and intra-abdominal infections (Mezzatesta et al., 2012; Davin-Regli et al., 2019). The accurate identification of species and subspecies in the genus *Enterobacter* has been a challenge due to the large variations in phenotypic and genotypic characteristics (Wu et al., 2021). Clinical microbial labs frequently fail to detect *Enterobacter* species, making it difficult to establish a clear correlation between specific species with antimicrobial resistance and clinical impact (Mezzatesta et al., 2012). Recently, *Enterobacter* taxonomy has been greatly improved because of the application of whole-genome sequencing (WGS) (Zong et al., 2021). To date, 24 species with known names and 21 taxa (genomospecies) without assigned names are designed in *Enterobacter* (Feng et al., 2021). Among them, a novel species of *E. chengduensis* was redefined through phenotypic and genotypic characteristics in 2019 (Wu et al., 2019). The *E. chengduensis* strain reported in this paper was recovered from a human blood sample in China in 2015. The whole genome sequence of this type strain exhibits an average nucleotide identity ranging from 80.48% to 93.34% when compared to the type strains of all recognized *Enterobacter* species. It can also be distinguished from all recognized *Enterobacter* species by its ability to ferment d-sorbitol, l-rhamnose and melibiose but with a negative Voges–Proskauer reaction. However, the epidemiological characteristics and molecular features of *E. chengduensi*s haven’t been reported since its identification and remain largely unknown.

Carbapenems serve as the cornerstone in the therapeutic arsenal against multidrug-resistant *Enterobacter* infections (Zhang et al., 2018). However, the escalating resistance to carbapenems within *Enterobacter spp.*, predominantly attributed to the acquisition of genes encoding carbapenemase enzymes (such as KPC, NDM, and VIM), has substantially undermined the efficacy of clinical antimicrobial treatment options (Nordmann and Poirel, 2019; Zong et al., 2021). Further investigations into the transmission mechanisms of carbapenemase-encoding genes will aid us in deciphering and limiting the spread of these genes in *Enterobacter*.

In our previous multicenter study (Zhu et al., 2022), we have preliminarily identified four clinical *bla*_NDM–1_-carrying *E. chengduensi*s strain. Notably, among 98 *Enterobacter* strains, *E. chengduensis* ranked third in terms of its prevalence. To provide valuable insights on this rarely reported *Enterobacter* species, and clarify the potential transmission mechanisms of carbapenemase-encoding genes, we conducted a detailed genomic study on these four *E. chengduensi*s strains. Herein, average nucleotide identity (ANI) and *in silico* DNA–DNA hybridization (isDDH) was used to identify the precise species of strains. The clonal relatedness of four *E. chengduensis* strains and additional 15 ones from NCBI were examined through phylogenetic analysis. The molecular features of *E. chengduensis* and genetic structure of *bla*_NDM–1_-carrying IncC plasmids were characterized through genomic annotation and analysis.

## Materials and methods

### Stain collection and antimicrobial susceptibility

Four carbapenem-resistant *Enterobacter* strains causing nosocomial infections were collected from seven tertiary health care hospitals in different provinces or cities in China between January 2017 and March 2021. Detailed bacterial strain collection was described in our previous study (Zhu et al., 2022). Clinical data were obtained from medical chart using the same standardized questionnaire in each hospital.

### Whole-genome sequencing (WGS)

Genomic DNA of carbapenem resistant *Enterobacter* strains were prepared using the Omega Bio-Tek Bacterial DNA Kit (Doraville, GA, USA). The extracted DNA was detected by the agarose gel electrophoresis and quantified by Qubit (Thermo Scientific). Sequencing libraries were generated using NEBNext Ultra DNA Library Prep Kit (NEB, USA) following manufacturer’s recommendations and index codes were added to attribute sequences to each sample. At last, PCR products were purified (AMPure XP system) and libraries were analyzed for size distribution by Agilent2100 Bioanalyzer and quantified using real-time PCR. Whole-genome sequencing was performed using the Illumina NovaSeq 6000 platform (Illumina Inc., San Diego, CA, USA) with the 350 bp paired-end protocols. Reads were filtered using *fastP* 0.17.1, and evaluated through FastQC 0.9.1. The above methods can ensure the quantity and quality of DNA data. The qualified data were then *de novo* assembled to contigs using *SPAdes* 3.11. Further plasmid assembly was obtained by mapping contigs on reference plasmid sequence (> 99% identical), checking overlapping paired ends and gap closure by PCR and Sanger sequencing.

### Species identification

Precise species identification was performed by calculating the pairwise isDDH and ANI value between the genome sequence of the query strain and the type strains of *Enterobacter* spp. described recently (Zong et al., 2021). The pairwise ANI with a ≥ 96% cutoff and isDDH with a ≥ 70.0% cutoff were used for precise species identification as previously suggested (Meier-Kolthoff et al., 2013; Rosselló-Móra and Amann, 2015). The isDDH analysis was performed by calculating identities/HSP length value through *GGDC* 3.0 (Meier-Kolthoff et al., 2022), while ANI analysis was performed by using *OAT* 0.93.1 (Lee et al., 2016).

### Genome analysis

Open-reading frames (ORFs) and pseudogenes were predicted using *RAST* 2.0 (Brettin et al., 2015) combined with *BLASTp/BLASTn* searches. Multilocus sequence typing (MLST) as well as annotation of resistance genes, mobile elements and other features were carried out using online databases including *PubMLST* (Jolley et al., 2018), *CARD* (Alcock et al., 2020), *ResFinder* (Bortolaia et al., 2020), *PlasmidFinder* (Carattoli et al., 2014), *ISfinder* (Siguier et al., 2006), and *INTEGRALL* (Moura et al., 2009). Multiple and pairwise sequence comparisons were performed using *BLASTn*. Gene organization diagrams were drawn through scripts from *Danmel* (Wang et al., 2022), and displayed using *Inkscape* 1.0.^1^

### Phylogenetic analysis

Zong et al. collected 4899 high-quality *Enterobacter* genome sequences within GenBank and carried out the precise species identification (Zong et al., 2021). Using the method described in this document, we further determined publicly available 15 *E. chengduensis* genome sequences within GenBank (excluding low-quality and duplicated sequences; accessed by January 19, 2022).

A phylogenetic analysis was performed on 19 identified *E. chengduensis* strains, including 15 strains from NCBI (Supplementary Table 1), and the four strains from this study. The genome sequences of *E. chengduensis* were aligned to the complete chromosome sequence (GenBank accession number CP004345) of *E. chengduensis* strain WCHECh050004, and the sequence of *E. cloacae* strain ATCC 13047 (GenBank accession number NC_014121) was used as an out group. The core single nucleotide polymorphisms (SNPs) were identified by *Mummer* 3.25 (Delcher et al., 2003). A maximum-likelihood phylogenetic tree was constructed using *MEGAX* 10.1.8 (Kumar et al., 2018) based on the core SNPs with a bootstrap iteration of 1000, and displayed using iTOL.^2^

### Conjugal transfer

We conducted conjugal transfer experiments between four *E. chengduensis* strains (HD5030, HD7411, HD7423 and HD7427) and the recipient *E. coli* J53 strain (NaN3-resistant) by combining mid-log-phase cultures of the donor and recipient (in a 1:10 donor to recipient ratio). Transconjugants were selected on plates with 2 μg/ml meropenem and 200 μg/ml NaN_3_. The conjugants were then confirmed by PCR for *bla*_NDM_ as previously described (Nordmann et al., 2011).

### Growth assay

Strains were grown overnight in 3 ml of LB with shaking (200 rpm) at 37°C, and then diluted to an OD600 of 0.25. 2 μl of the solution was added to 200 μl of LB in a 96-well plate in triplicate. Culture densities were determined every 10 min by measuring the OD600 for 16 h with shaking (200 rpm) at 37°C via FLUOstar Omega (BMG Labtech, Germany). Growth curves were estimated via *GraphPad Prism* 5.0 (GraphPad Software, Inc.) using two-way analysis of variance (ANOVA), and *p*-values < 0.05 were reported as statistically significant.

### Nucleotide sequence accession numbers

The draft genome sequences of four *E. chengduensis* strains HD5030, HD7411, HD7423 and HD7427 were submitted to GenBank under BioProject PRJNA824645. The sequences of four mapped plasmids were submitted to GenBank under accession numbers ON209151-ON209154.

## Results

### Emergence of *E. chengduensis* strains

We identified four non-duplicate *E. chengduensis* strains (Supplementary Table 2) from the 92 *Enterobacter* strains described in our previous study (Zhu et al., 2022). The four strains (HD5030, HD7411, HD7423 and HD7427) were collected from the emergency intensive care unit (ICU) or gastroenterology department in a tertiary care hospital in Southwest China (Supplementary Table 2). All strains exhibited resistance to cephalosporins (ceftazidime, cefepime, cephalothin, cefazolin, ceftriaxone etc.), carbapenems (meropenem, imipenem, ertapenem), piperacillin/tazobactam, and fluoroquinolones (ciprofloxacin, levofloxacin, moxifloxacin), while they were susceptible to amikacin, sulfamethoxazole. Antibiotic resistance gene screening through *ResFinder* revealed that all four *E. chengduensis* strains carried the *bla*_NDM–1_ gene (Supplementary Table 3).

### MLST and phylogenetic analysis of *E. chengduensis* strains

**FIGURE 1:**
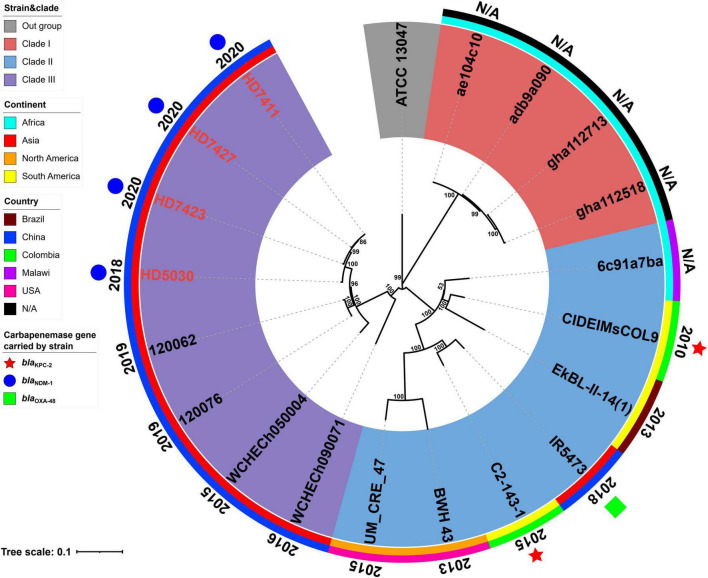
A Maximum-likelihood phylogenetic tree of ST414 *Enterobacter chengduensis* strains. A total of 19 ST414 *E. chengduensis* strains (including four reported in this study, and 15 from NCBI) were performed with phylogenetic analysis, while *E. cloacae* strain ATCC 13047 (GenBank accession number NC_014121) was used as the out group. Bar corresponds to scale of sequence divergence.

The results of MLST analysis showed that all 19 *E. chengduensis* strains analyzed in this study (including 15 ones from NCBI and four ones reported in this study) belonged to ST414 (Supplementary Tables 1, 2). A total of 2066 core genome SNPs were identified from these 19 strains and were used to construct a maximum-likelihood phylogenetic tree (Figure 1). The 19 strains were divided into three clades (clade I-III). A total of seven strains carried the carbapenemase-encoding gene, of which two strains from Colombia carried the *bla*_KPC–2_ gene, while five strains from China carried the *bla*_OXA–48_ gene (*n* = 1) and *bla*_NDM–1_ gene (*n* = 4). All four *bla*_NDM–1_-carrying strains belonged to clade III, while the other three belonged to clade II.

The core SNPs of 19 ST414 *E. chengduensis* strains were further pairwise compared to determine their clonal relatedness (Supplementary Table 4). In the nine strains from China, there were 5 to 592 SNP differences, suggesting that they did not belong to a single clone. However, the three strains in this study, HD7411, HD7423 and HD7427, only had only 5–10 SNP differences. Furthermore, these three strains were collected from different patients in two different wards in a hospital between April and June 2020. The hospitalization times of the three patients infected with these three strains overlapped, and the collection interval of these three strains was within one month (Supplementary Figure 1). These results suggest the potential intrahospital clonal transmission of ST414 *E. chengduensis* strains. Additionally, SNP comparison also showed that two *bla*_KPC–2_-carrying *E. chengduensis* strains did not belong to a single clone (having 302 SNPs difference).

### Plasmid carrying the *bla*_NDM–1_ gene

**FIGURE 2:**
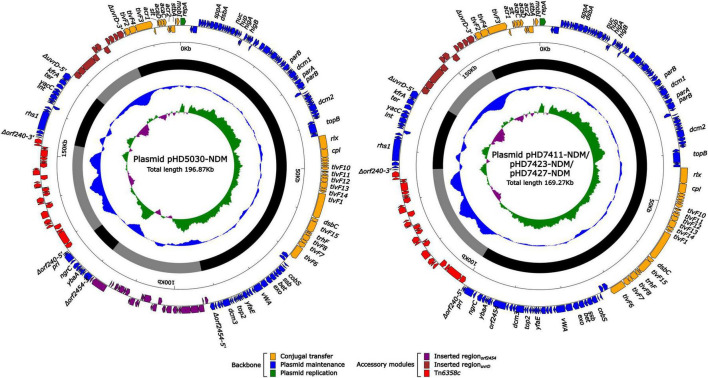
Schematic diagram of IncC-type plasmids assembled in this study. Genes of different functions are denoted by arrows and presented in various colors. The circles show (from outside to inside): predicted coding sequences, scale in 10 kb, backbone (black) and accessory module (gray) regions, GC content and GC skew [(G–C)/(G + C)].

**FIGURE 3:**
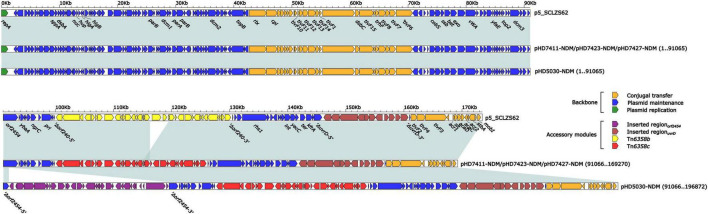
Linear comparison of IncC-type plasmids analyzed in this study. Genes are denoted by arrows. Genes, mobile genetic elements and other features are colored based on function classification. Shading regions denote homology of two plasmids (light blue: ≥ 99% nucleotide identity). The accession number of plasmid used as reference is CP082173.

**FIGURE 4:**
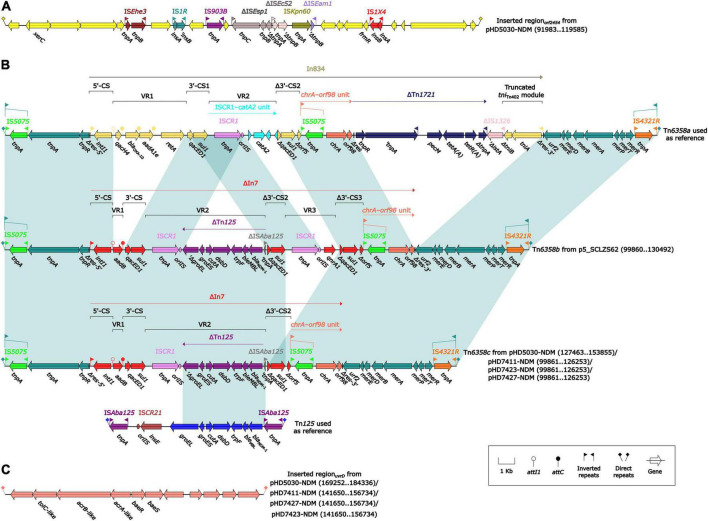
Organization of **(A)** inserted region_*orf2454*_, **(B)** *bla*_NDM_ regions, and **(C)** inserted region_*uvrD*_ from *bla*_NDM_-harboring plasmids. Genes are denoted by arrows. Genes, mobile genetic elements and other features are colored based on their functional classification. Shading denotes regions of homology (light blue: ≥ 99% nucleotide identity). The accession number of Tn*6358a* and Tn*125* used as reference are JX141473 and JN872328, respectively.

The *bla*_NDM–1_ genes of four strains were all carried by IncC plasmids (Table 1). Each IncC plasmid could be divided into the backbone and two or three accessory modules, which resulted from exogenous DNA region insertion at different sites of the backbone (Figure 2). The backbones of the four plasmids were almost identical (Table 1) to the IncC reference plasmid p5_SCLZS62 (GenBank accession number CP082173), while these five plasmids were mainly different in accessory modules (Figures 3, 4). First, compared with p5_SCLZS62 and the other three plasmids, the plasmid pHD5030-NDM was interrupted by a 27.6-kb inserted region*_orf2454_* containing several inserted sequences (IS) (Figure 4A). Second, the four plasmids and reference plasmid p5_SCLZS62 all carried unit transposon Tn*6358* variants (Figure 4B). Among them, p5_SCLZS62 harbored Tn*6358b*, whereas the four plasmids in this study harbored Tn*6358c*. Tn*6358b* and Tn*6358c* were inserted within the *orf240* gene from the backbone of IncC plasmids and were flanked by 5-bp direct repeats (DRs). Moreover, Tn*6358b* and Tn*6358c* differed from Tn*6358a* (GenBank accession number JX141473) by exchanging integron In834 for truncated integron In7. However, truncated integron In7 from Tn*6358b* harbored three resistance loci: gene cassette array (GCA) *aadB* (VR1: variable region 1), IS*CR1*–truncated Tn*125* (carrying *bla*_NDM–1_) region (VR2), and IS*CR1*–*qnrA1* unit (VR3), while truncated integron In7 from Tn*6358c* further lost a 4.3-kb region including the VR3, a truncated *qacED1* gene and a *sul1* gene. In addition, the four plasmids all harbored the 15.1-kb inserted region*_uvrD_* (Figure 4C), which was inserted within *uvrD* from backbone of IncC plasmid and was flanked by 6-bp DRs. The inserted region*_uvrD_* carried a predicted efflux RND transporter (*acrAB*-*tolC*-like) and its function was not clear.

**TABLE 1:** Information of IncC-type plasmids assembled in this study.

Plasmid	Total plasmid		Backbone region		Location of *bla*_NDM–1_
	Length (bp)	Comparison with reference plasmid* [coverage + identity (%)]	Length (bp)	Comparison with reference plasmid [coverage + identity (%)]	
pHD5030-NDM	196,872	99 + 99.99	127,803	100 + 99.99	Tn*6358c*
pHD7411-NDM	169,270	99 + 99.99	127,803	100 + 99.99	
pHD7423-NDM	169,270	99 + 99.99	127,803	100 + 99.99	
pHD7427-NDM	169,270	99 + 99.99	127,803	100 + 99.99	

*The reference plasmid was p5_SCLZS62 (GenBank accession number CP082173).

Conjugal transfer experiment showed that all four *bla*_NDM–1_-carrying IncC-type plasmids could be transferred from host strains into the recipient *E. coli* J53. To assess the impact of the IncC-type plasmid on the growth of the strain, the growth of *E. coli* J53 was compared with the growth of its corresponding transformant carrying the IncC-type plasmid pHD5030-NDM. The growth curves of *E. coli* J53 and J53/pHD5030-NDM showed no significant difference (*p* > 0.05; Supplementary Figure 2). These results indicated that the IncC-type plasmid was self-transmissible, and had little impact on the growth of the *E. coli* J53 strain in LB.

## Discussion

Studies on epidemiological characteristics and molecular features in *E. chengduensis* are very limited since this species was first reported in 2019 (Wu et al., 2019). Only one review paper identified 12 *E. chengduensis* strains from 4899 *Enterobacter* genomes (accessed by September 30, 2020) from the NCBI database through isDDH analysis (Zong et al., 2021). However, this review paper didn’t provide a detailed description of the characteristics of *E. chengduensis*. In order to clarify epidemiological characteristics and molecular features of *E. chengduensis*, we retrieved 15 *E. chengduensis* genomes from NCBI (accessed by January 19, 2022; Supplementary Table 1), and included four clinical *E. chengduensis* strains (Supplementary Table 2) from 98 *Enterobacter* strains collected in our previous study (Zhu et al., 2022) for further research. Interestingly, MLST analysis showed that all 19 *E. chengduensis* strains belonged to ST414. These results suggested that the current spread of *E. chengduensis* was still uncommon, and ST414 might be the predominant ST in *E. chengduensis*.

Nevertheless, the raise of carbapenem-resistance in *E. chengduensis* strains may spur the further dissemination of this species, and cause a significant threat to public health, which has been demonstrated in other *Enterobacter* species (Nordmann and Poirel, 2019; Zong et al., 2021). Among 15 *E. chengduensis* strains from NCBI, only three strains carried carbapenemase-encoding genes, including two carrying *bla*_KPC–2_ and one carrying *bla*_OXA–48_. However, all four *E. chengduensis* strains reported in this study carried *bla*_NDM–1_ gene (Supplementary Table 3). Phylogenetic analysis showed that these four *E. chengduensis* strains have relatively close homology (Figure 1). Furthermore, potential intrahospital clonal transmission of three strains among them was also detected (Supplementary Table 4 and Supplementary Figure 1). In view of this, *bla*_NDM–1_-carrying *E. chengduensis* should be closely monitored in China.

Horizontal transfer is one of the main mechanisms for carbapenemase-encoding gene transfer. In this study, we found that the *bla*_NDM–1_-harboring IncC-type plasmid was the only carbapenemase-encoding plasmid detected in the strains sequenced herein (Figure 2 and Table 1). These four plasmids shared highly identical backbone region (Figure 3 and Table 1), demonstrating their high level of homology. The IncC-type plasmid was conjugative, which was confirmed in this study and previous reports (Ambrose et al., 2018; Zheng et al., 2020; Fernandez et al., 2022). From the limited data in this study, *E. chengduensis* does not appear to be the natural host of the *bla*_NDM_ gene. Thus, the self-transferability and low fitness cost (Supplementary Figure 2) of the *bla*_NDM–1_-harboring IncC-type plasmid may in part contribute to the emergence of *bla*_NDM–1_-carrying *E. chengduensis*. Additionally, IncC-type plasmid and its derived hybrid plasmid have been reported as an important vector for novel resistance genes, such as *bla*_VMB–1_ (Zheng et al., 2020), and *tmexCD3-toprJ3* (Hirabayashi et al., 2021), which is cause for concern.

All *bla*_NDM–1_ genes reported in this study were carried by truncated Tn*125* (Figure 4B). Tn*125*, a composite transposon bordered by two copies of IS*Aba125* (Poirel et al., 2012), plays a critical role in the initial spread of the *bla*_NDM_ gene to plasmid (Acman et al., 2022). The truncated Tn*125* element in this study was captured by the truncated integron In7 as a variable region, and subsequently integrated into the unit transposon Tn*6358* (Wang et al., 2022). Although the mobility of Tn*6358* has not been experimentally confirmed, the genetic element that can integrate various resistance genes after sophisticated events of transposition and homologous recombination needs to be cautious.

This study has some limitations. Firstly, the sample size of *E. chengduensis* in this study was small. The small sample size was in part because we focused on non-duplicated and high-quality sequences. Nevertheless, this dataset represents relatively comprehensive *E. chengduensis* genomic resources currently available and the data provide valuable insights on this rarely reported *Enterobacter* species. Secondly, our analysis on clonal relatedness of ST414 *E. chengduensis* strains, and function of IncC plasmid are not comprehensive, which necessitate further in-depth investigations.

In conclusion, this study reports the first emergence and potential clonal transmission of *bla*_NDM–1_-carrying *E. chengduensis*. ST414 might be the predominant ST in *E. chengduensis*. The IncC-type plasmid serves as the main vector of *bla*_NDM–1_ in this rarely reported *Enterobacter* species. Further surveillance should be advocated to monitor the dissemination of ST414 *E. chengduensis* and *bla*_NDM–1_-harboring IncC-type plasmids in China.

## Data Availability

The datasets presented in this study can be found in online repositories. The names of the repository/repositories and accession number(s) can be found in the article/Supplementary material.

## Footnotes

1
https://inkscape.org/

2
https://itol.embl.de

## Supplementary material

Supplementary Figure 1 Schematic diagram of hospitalization of patients. Blue lines represent admission date of patients; green lines represent discharge date of patients; and red lines represent collection date of *Enterobacter chengduensis* strains.

Supplementary Figure 2 Growth curves of J53 and its transformant J53/pHD5030-NDM. Optical densities of *E. coli* J53 and its transformant J53/pHD5030-NDM in LB without antibiotic.
