# Supplementary figures and images for: Emergence of blaNDM–1-carrying Enterobacter chengduensis in China
Source: Front Microbiol. 2024 Aug 14;15:1404996. doi: 10.3389/fmicb.2024.1404996 (PMC11350614; doi:10.3389/fmicb.2024.1404996)

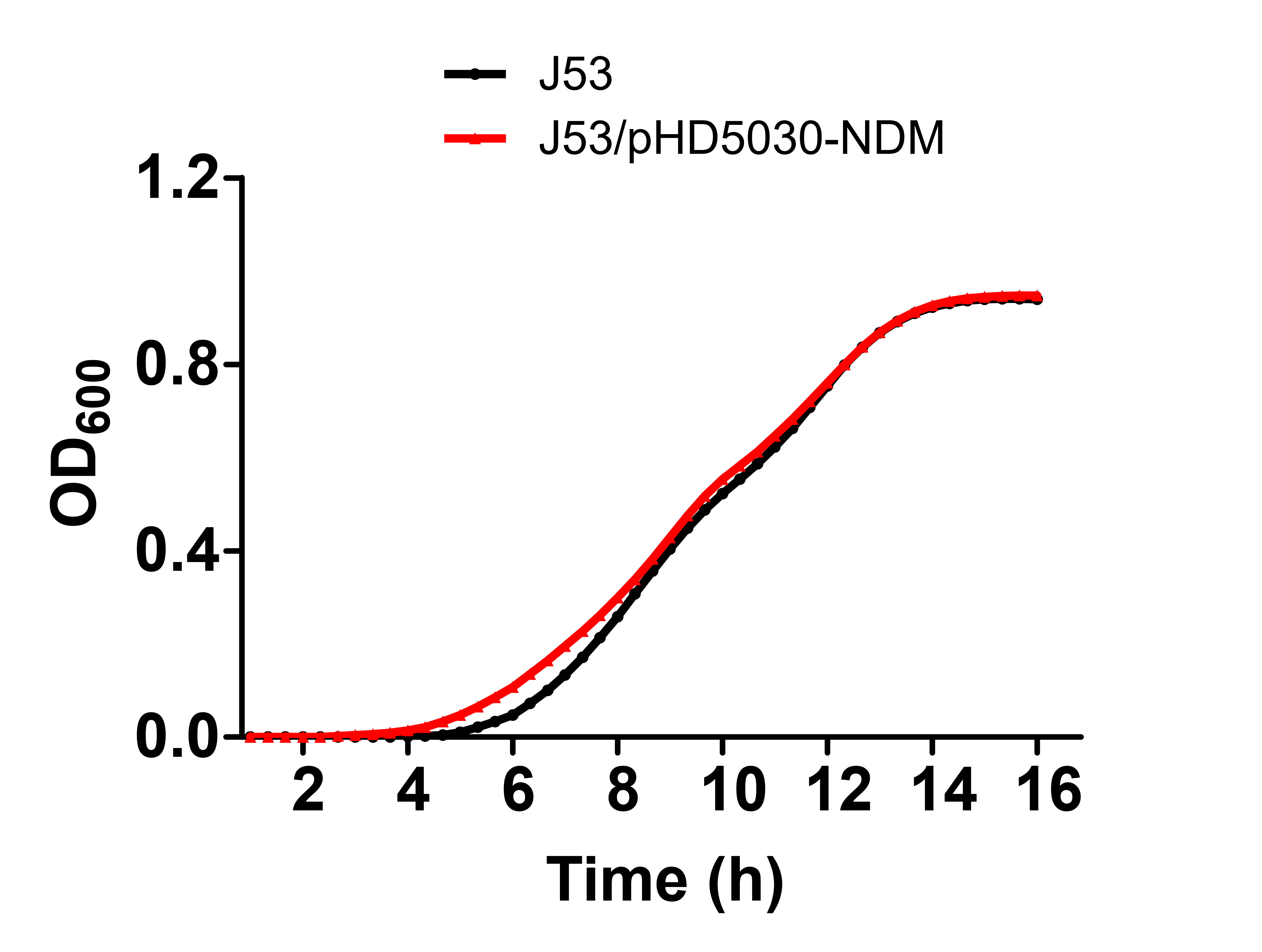

Supplement: Supplementary Figure 2 — Growth curves of J53 and its transformant J53/pHD5030-NDM. Optical densities of E. coli J53 and its transformant J53/pHD5030-NDM in LB without antibiotic. [file Image_2.TIF]
